# Predicting yellow rust in wheat breeding trials by proximal phenotyping and machine learning

**DOI:** 10.1186/s13007-022-00868-0

**Published:** 2022-03-15

**Authors:** Alexander Koc, Firuz Odilbekov, Marwan Alamrani, Tina Henriksson, Aakash Chawade

**Affiliations:** 1 Department of Plant Breeding, Swedish University of Agricultural Sciences, P.O. Box 190, SE-234 22 Lomma, Sweden; 2 Lantmännen Lantbruk, SE‐268 81 Svalöv, Sweden

**Keywords:** High-throughput phenotyping, Plant breeding, Yellow rust, Field phenotyping, Spectral vegetation index, Low-cost phenotyping, Winter wheat, Disease resistance

## Abstract

**Background:**

High-throughput plant phenotyping (HTPP) methods have the potential to speed up the crop breeding process through the development of cost-effective, rapid and scalable phenotyping methods amenable to automation. Crop disease resistance breeding stands to benefit from successful implementation of HTPP methods, as bypassing the bottleneck posed by traditional visual phenotyping of disease, enables the screening of larger and more diverse populations for novel sources of resistance. The aim of this study was to use HTPP data obtained through proximal phenotyping to predict yellow rust scores in a large winter wheat field trial.

**Results:**

The results show that 40–42 spectral vegetation indices (SVIs) derived from spectroradiometer data are sufficient to predict yellow rust scores using Random Forest (RF) modelling. The SVIs were selected through RF-based recursive feature elimination (RFE), and the predicted scores in the resulting models had a prediction accuracy of *r*_*s*_ = 0.50–0.61 when measuring the correlation between predicted and observed scores. Some of the most important spectral features for prediction were the Plant Senescence Reflectance Index (PSRI), Photochemical Reflectance Index (PRI), Red-Green Pigment Index (RGI), and Greenness Index (GI).

**Conclusions:**

The proposed HTPP method of combining SVI data from spectral sensors in RF models, has the potential to be deployed in wheat breeding trials to score yellow rust.

**Supplementary Information:**

The online version contains supplementary material available at 10.1186/s13007-022-00868-0.

## Introduction

Plant breeding for disease resistance remains an important tool in reducing crop yield loss due to pathogens. Resistance breeding relies on screening of large populations under field conditions, with the purpose of identifying superior parents for crossing into progeny, or novel genetic sources for resistance. This process typically relies on visual disease scoring of hundreds to thousands of plots performed by human raters. While the method of visual scoring has served plant breeders well, it is a time-intensive process, and the quality and reliability of the collected visual disease scores is dependent on the experience and training of the individual raters. While increasing the number of raters alleviates the problem of low throughput, the subjectivity of each rater makes it difficult to compare and analyze the resulting scores. This problem persists even when comparing data from experienced raters [1]. In short, the time-intensive and subjective nature of visual scoring puts a limit on the scale of breeding trials and accuracy of the visual disease scores. The research and development of field high-throughput plant phenotyping (HTPP) aims to resolve this bottleneck and accelerate plant breeding, by enabling rapid, cheap and scalable phenotyping methods [2–4]. There are several factors to consider, if a proposed HTPP method is to replace the golden standard of visual disease scoring. HTPP methods should have an acceptable selection accuracy compared to the visual scores. The measurements obtained through the method should have an acceptable heritability/repeatability across different environments and be robust to variation caused by genotypic diversity. Finally, any proposed method should strive to be affordable and be easy to implement, to facilitate adoption by plant breeders [5].

Wheat yellow rust—also called wheat stripe rust—is caused by the fungal pathogen *Puccinia striiformis *f. sp. *tritici.* Yellow rust has been a major wheat disease for centuries and even today the massive crop yield loss caused by yellow rust in wheat cultivation makes it the economically most destructive rust disease [6–8]. The disease symptoms include degradation of leaf chlorophyll, followed by the formation of stripes of yellow to orange urediniospores along the axis of the leaf [6]. To date, the most effective way of controlling yellow rust is developing resistant cultivars.

A number of studies have investigated potential methods for detecting and quantifying yellow rust and other foliar diseases in wheat. One of these studies investigated ten widely-used spectral vegetation indices (SVI) to discriminate between rust-infected and healthy wheat leaves [9]. The authors found that detection of yellow rust was possible using SVIs sensitive towards changes in leaf pigment concentrations, indicating that spectral sensors can be used to detect yellow rust under controlled conditions. In the same vein, a different study developed novel three-band SVIs for quantifying and detecting yellow rust based on the Anthocyanin Reflectance Index (ARI) and Photochemical Reflectance Index (PRI) and compared those against previously published SVIs [10]. Prediction of yellow rust under field conditions has been shown to be possible using a hyperspectral imaging sensor on an unmanned aerial vehicle (UAV) and a ground-based phenotyping platform [11]. The authors used a combined approach of supervised classification of pixels and regression, to investigate how well the classifier predictions related to visual scores of yellow rust in individual wheat plots. Both the ground-based and UAV approach resulted in reporting a high yellow rust prediction accuracy. An earlier study [12] evaluated the identification of the disease progression stages of another wheat foliar disease, Septoria tritici blotch (STB), using sensor phenotyping and machine learning under greenhouse conditions. A training and validation set of 10 winter wheat genotypes were inoculated with *Zymoseptoria tritici* suspension and assessed at multiple time points for disease severity. A second population counting two cultivars was designated as a test set and evaluated for STB. Proximal phenotyping of both populations involved collecting data on Photosystem II quantum-yield using an active light fluorometer, spectral reflectance data (350–1150 nm) using a spectroradiometer, and finally leaf temperature data with an infrared thermometer. Random forest models in combination with recursive feature selection were successfully used for selecting and evaluating predictors for both chlorosis and necrosis [13] developed a promising time-resolved spectral method for quantifying STB severity in winter wheat under field conditions. The authors argued that spectral features estimated at single time points lack specificity to disease effects across time and are not robust to changes in reflectance caused by nuisance factors and environmental conditions. The proposed method uses spectral-temporal features based on two spectral vegetation indices tracking relative changes in spectra over time and achieving a prediction accuracy of Pearson’s r = 0.53 for correlation between observed and predicted STB severity in a validation population of 330 winter wheat genotypes. These studies show that detection of foliar diseases in wheat is possible using high-throughput phenotyping methods.

The main objectives of this study were to automate disease scoring of yellow rust in a large plant breeding field trial, by combining high-throughput phenotyping data from low-cost proximal sensors with machine learning. Sensor data obtained from imaging and spectral sensors was evaluated for prediction of yellow rust using data collected, with the help of a ground-based phenotyping platform in two winter wheat populations. The populations were grown in the winter wheat growing seasons 2019/2020 and comprised: (1) a diverse panel of Nordic and Baltic winter wheat landraces and cultivars and (2) advanced winter wheat breeding lines. A random forest based approach was used to relate visual disease scores to the HTPP data. The performance of the models was evaluated by assessing the prediction accuracy within the time points at which data was collected.

## Materials and methods

### Plant material and experimental setup

Winter wheat field trials were conducted in a field in Southern Sweden (55°54′34.1′′N 13°09′30.4′′E). The plant material in both trials consisted of two winter wheat populations sown in the winter wheat growing season of 2019/2020: a panel of 211 genotypes including cultivars and landraces selected from the Nordic Genebank (NordGen), and Baltic and Swedish cultivars (Genebank set). The second population (Breeding set) consisted of 325 advanced-stage F_5_ crosses from an ongoing private wheat breeding program (Lantmännen Lantbruk, Svalöv, Sweden). Disease and HTPP data were collected in both populations on two dates in July: 2020-07-02 and 2020-07-09.

### Disease assessments

Disease score data for yellow rust was collected by visual assessment in both populations by the same rater. The severity of diseases was rated as an average score across the whole plot, on a scale of 1–9, where 1 and 9 corresponds to 0–10% and 90–100% of the plot covered by disease respectively. In this study, we relied on natural disease pressure in the populations.

### Phenocart and digital sensors

HTPP data was collected from a ground-based phenotyping platform (phenocart), which was used to move a set of sensors over individual wheat plots in the field. For more details about the exact dimensions and setup of the phenocart, and how the phenocart is used in combination with sensors for phenotyping, please refer to Sect. 2.1 in [14].

The sensors used in this study to phenotype the plant material can be categorized into two types: imaging and spectral sensors. The imaging sensors consisted of two consumer-grade digital single- lens reflectance (DSLR) cameras (Canon 1300D, Canon, USA). The first camera (NDVI camera) was modified (LifePixel Infrared, USA) to capture spectral data in the blue, green and near-infrared (NIR) range. A modified version of the normalized difference vegetation index (NDVI) [15] was estimated using data from the Blue and NIR bands (BNDVI), according to the following formula:

$$\text{BNDVI} = \frac{{\text{NIR}}-{\text{Blue}}}{\text{NIR+Blue}}$$

The second camera was left unmodified and was used for regular Red–Green–Blue (RGB) image capture. The spectral sensors included two Apogee SS-110 Field Spectroradiometers (Apogee Instruments, Inc., Logan, Utah, USA) used to capture non-imaging hyperspectral data in the spectral range of 340–820 nm, with a spectral band resolution of 1 nm. The spectral sensors were set up with one sensor mounted pointing directly up at the sky at the top of the phenocart with a field of view (fov) attachment of 180° for measuring incoming radiation. The other sensor was mounted at the front of the phenocart, angled 45° down towards the canopy at a fov of 15° for measuring the radiation reflected from the wheat canopies. Both spectral sensors were synchronized and operated in continuous reflectance data capture mode using the Apogee SpectroVision software (version 1.03.004, Apogee Instruments, Inc., Logan, Utah, USA). The process of synchronizing both spectral sensors involved setting the white reference of the canopy-facing spectroradiometer against an Apogee AS-004 white reflectance standard plate (Apogee Instruments, Inc., Logan, Utah, USA). This sensor synchronization process was repeated every 40th plot in 2020. Each spectral sample was averaged over five spectral scans and the integration time of both sensors was set depending on prevailing light conditions.

### Image data processing

The initial processing of image data from both RGB and NDVI cameras was done using the RawTherapee software (version 5.6), where the exposure in each image was adjusted using RawTherapee’s exposure auto-adjust function. The white balance of each image was adjusted against the grey card in the images. The center of each plot was cropped from each image into a reduced size (1000 × 800 px) image. The cropped images from the RGB camera were processed into digital green biomass estimates by using a previously published pipeline [16]. Briefly, this pipeline relies on hue, saturation, and value (HSV) thresholding to identify and quantify green vegetation in RGB images. The cropped images from the NDVI camera were processed into NDVI estimates using a custom R script. The R script calculated the NDVI value for each pixel in the input image. A single NDVI value was estimated for each image by calculating the mean NDVI over all pixels in the input image. Only pixels over an NDVI threshold of 0.2 were considered when calculating the mean, to remove any soil or background data.

### Spectral data processing

The processing of spectroradiometer data progressed in three steps: (1) calculation of canopy spectral reflectance (2) quality control performed on the calculated reflectance data and (3) calculation of spectral vegetation indices (SVIs). Canopy reflectance was calculated at each measured wavelength by using a custom R script to calculate the fraction of reflected radiation according to the following equation:

$$R = \frac{{E_{{{\text{canopy}}}} }}{{E_{{{\text{reference}}}} }},$$

where $R$ is the fraction of reflected spectral radiation, ${E}_{\text{canopy}}$ and ${E}_{\text{reference}}$ are the raw digital spectral counts obtained from the canopy-facing and upward-facing spectroradiometers respectively. Quality controlled involved visual assessment of the resulting spectral data to remove bad data, such as for example spectral data collected from empty wheat plots. The processing of the data proceeded using version 1.0.3 of the hsdar R package [17], the spectra were visually inspected, with the result that data from spectral bands outside of the interval of 400–800 nm and inside the interval of 755–771 nm were removed due to poor signal-to-noise ratio [18]. The spectral data remaining after quality control was smoothed using a Savitzky–Golay smoothing filter with a filter length size of 11 bands and filter order set to third order polynomial, using the smoothSpeclib() function available in hsdar. The resulting cleaned spectral data was processed using hsdar and Specalyzer R packages [19] into 119 spectral vegetation indices (SVIs). For a full list of SVIs used in this analysis, please refer to [17, 19]. In addition to the indices available via hsdar and Specalyzer, we included two spectral vegetation indices developed for detecting and measuring yellow rust: SVI_YR [20] and SVI_PRI_YR [10].

### Training and evaluation of machine learning models

The data processing of the sensor data resulted in 119 HTPP predictors: two image-data based predictors and 117 SVIs. Automatic PCA-based outlier removal was performed as described earlier [19], and any rows with missing data were removed from the final data set. This yielded 439 and 505 observations for the 2020-07-02 and 2020-07-09 timepoints respectively. A correlation analysis was conducted in R to explore the linear association between these predictor variables and the disease scores at each time point. To this end, the Spearman’s correlation coefficient was computed for each predictor in association with the disease scores. Following the correlation analysis, the data at each timepoint was split into an 80/20 training and test data set, where the training data was used to perform recursive feature elimination and the test set was reserved for the validation of the final model trained based on the results of the feature elimination. Thus, a minimal set of predictors was established by performing supervised feature selection by recursive feature elimination with random forest (RF) regression, by adapting a protocol and associated code by [13]. The feature elimination was performed separately for each time point and proceeded by stepwise elimination of predictors from 119 predictors down to one, repeated 30 times. In each repeat, the previously defined training set was further split into an 80/20 training/test set. This reduced feature elimination training set was used to train random forest models, which were sued to compute variable importance and model performance metrics. Variable importance was used to rank the features at each step of the elimination, whereas model performance was assessed by calculating the root-mean-square error (RMSE) of prediction on both the feature elimination training and test data. This process was repeated 30 times, where the reduced training/test sets were resampled at each repeat. RF was used as the base-learner, where the number of trees was set to 1000 and ten-fold CV was used for model tuning. The elimination proceeded in one step from 119 variables to 100, from 100 to 30 by increments of five, and from 30 to 1 by increments of one. The resulting feature rankings and model performance metrics were averaged across all resamples into a robust estimate of variable ranking and model RMSE. The number of features in the final model was selected by plotting training and test set RMSE against the number of features in the feature elimination, the selection was made based on lowest test set RMSE and minimal number of predictors. The R packages caret [21] and ranger [22] were used to fit RF regression models for feature selection and the following model validation.

The final RF yellow rust prediction models were trained on the selected features extracted from the full training set. Model tuning and selection in caret was performed using ten-fold cross-validation. The predictive performance of each model was assessed by predicting yellow rust scores using the test set HTPP data. The performance for each model was computed by calculating RMSE and the Spearman's rank correlation coefficient between predicted and observed disease scores. As before, the validation was performed separately for each time points. The final model validation step was repeated by retraining the models the models without including the image-based predictors. The purpose of this was to assess the contribution of the image-based predictors to the model accuracy.

## Results

Yellow rust disease score assessments were collected in two winter wheat populations together with HTPP data from imaging and spectral sensors. A correlation analysis was performed to assess the linear association of the HTPP data with the disease score data. RF models were trained and evaluated with regards to prediction accuracy of yellow rust disease scores at specific timepoints. A variable importance analysis was performed to deduce which HTPP data and, by extension, which sensors were useful in predicting the disease outcome.

### Phenotypic characterization of disease infection

**Fig. 1:**
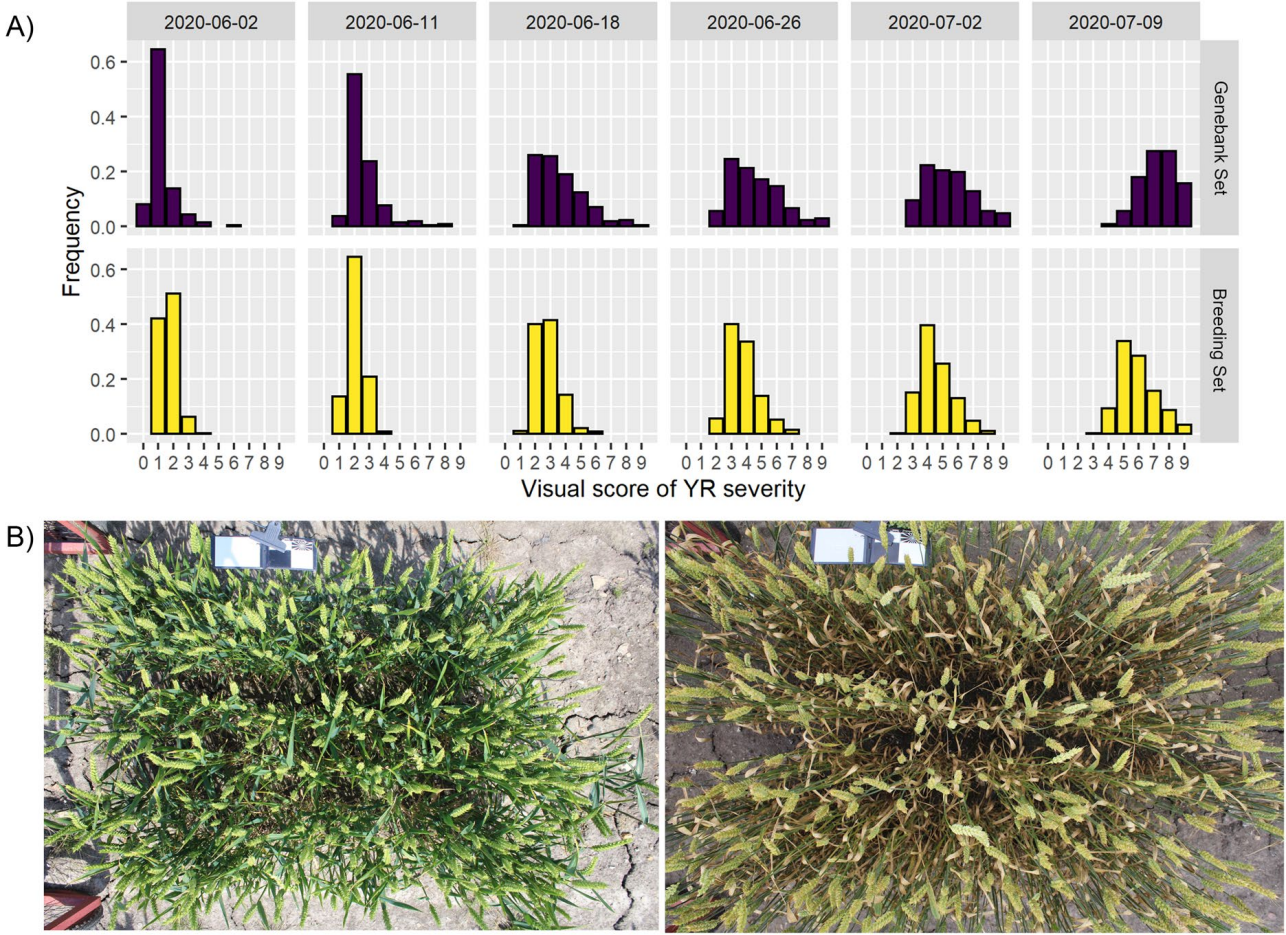
Yellow rust disease progression in the experimental populations. **a** Phenotypic distributions of yellow rust (*Puccinia striiformis*) severity in two winter wheat (*Triticum aestivum*) population: (Genebank set) A set of 211 genotypes including material from Nordic Genebank, Nordic and Baltic cultivars, and (Breeding set) a set of 325 advanced stage breeding F5 crosses. The disease observations were performed at six time points in the winter wheat growing season 2019/2020. **b** Two example images of a Healthy (left) and diseased (right) wheat plot

There was considerable development of natural yellow rust infection in both populations starting in the beginning of June. Toward the end of the season, most plots in both populations exhibited moderate to high yellow rust infection. Plots in the genebank population tended towards higher disease severity compared to the breeding material (Fig. 1).

### Correlation between sensor data and observed data of disease scores

**Fig. 2:**
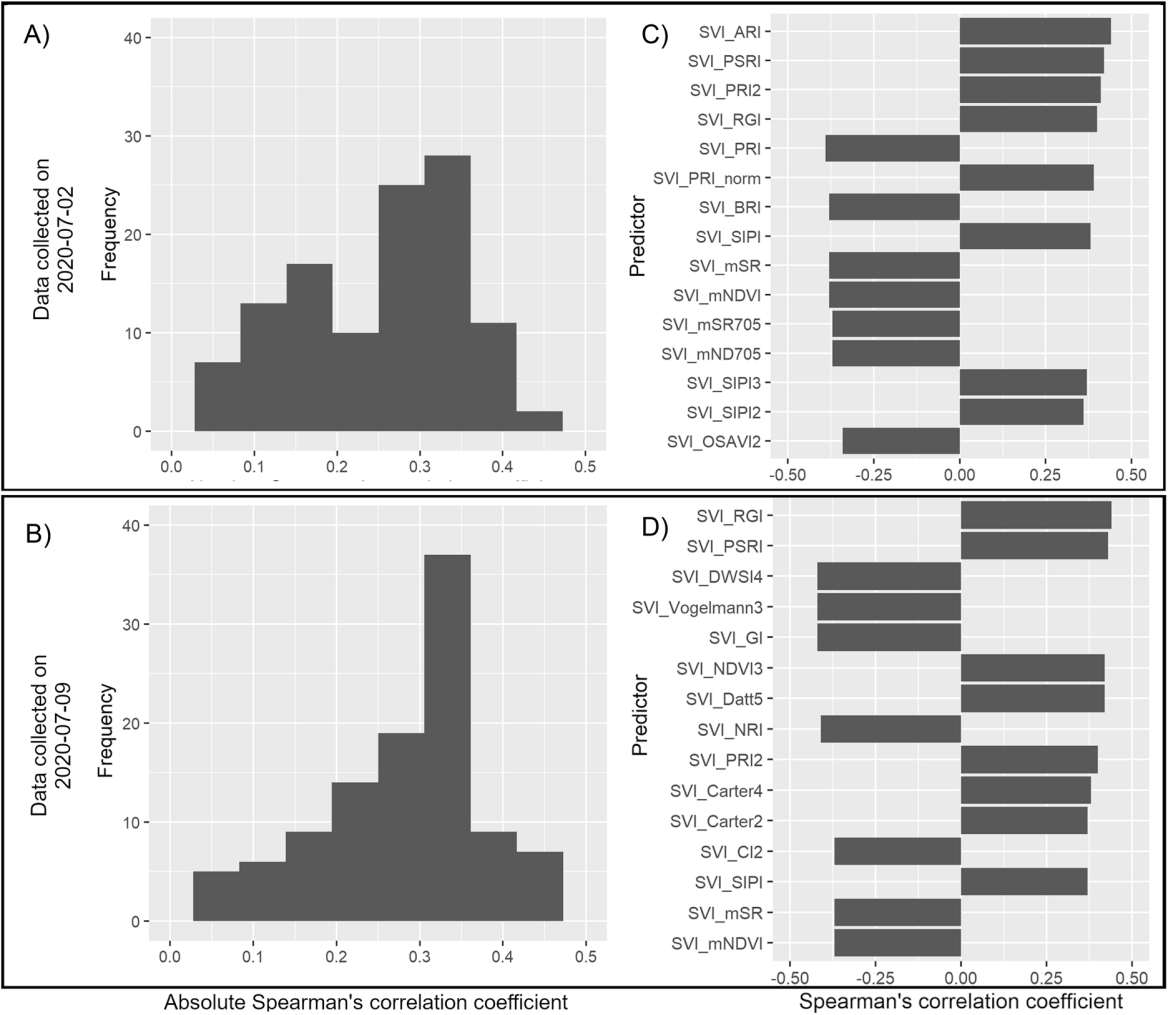
Correlation analysis between predictors and YR scores. Spearman’s correlation analysis between yellow rust disease scores and model predictor variables derived from high-throughput phenotyping (HTPP) data collected at two dates under field conditions. **a**, **b** Distributions of strength of association between HTPP predictors and disease scores measured in absolute correlation values. **c**, **d** Top 15 predictors ranked in descending order from top to bottom by their absolute correlation value with observed disease scores

A correlation analysis was performed on data from both time points of scoring to quantify the linear association between the predictor variables obtained from the HTPP data and the observed disease scores. The highest absolute correlation between HTPP predictors and disease data reached a value of ${r}_{abs}=0.43$ for data collected at 2020-07-02, and ${r}_{abs}=0.43$ for 2020-07-09. The distributions of the correlation and the ranking of most correlated predictors are shown in Fig. 2. The predictors showing the highest correlations with the disease score data were SVIs calculated from the spectroradiometer reflectance data. Conversely, the data obtained from imaging-sensors showed low to none correlation with the disease score data (Additional file 1: Table S1).

### Feature selection and variable importance

**Fig. 3:**
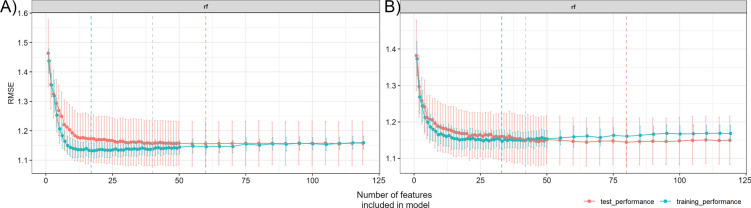
Recursive feature elimination of predictors. Results of recursive feature elimination in two datasets of yellow rust disease scores and high-throughput phenotyping data collected under field conditions. The dashed vertical lines indicate the selected optimal number of model predictors based on training (blue) and test set (red) Root Mean Square Error (RMSE), the grey dashed line indicates the number of features selected for the final model. **a** Disease score and HTPP data collected on 2020-07-02 (N = 351), **b** Disease and HTPP data collected on 2020-07-09 (N = 404)

Recursive feature elimination was performed separately for the two time points to obtain robust estimates of variable importance and to identify an optimal number of features for the disease prediction models. The final number of predictors for both models were selected by selecting for lowest number of predictors in combination with lowest test set root mean square error (RMSE). Both datasets saw little change in RMSE when decreasing the number of features from the 119 original features to 42 and 40 features for the 2020-07-02 and 2020-07-09 datasets respectively (Fig. 3). Whereas decreasing the number of features to beyond 42 features led to a steady increase in test set RMSE, with a steep increase below 10–12 predictors for both timepoints.

The selected predictors were similar in the data from the two time points (Table 1), with 23 features shared by the models at each time point. Among the top 10 predictors were variations of the photochemical reflectance index (PRI), together with the Plant Senescence Reflectance Index (PSRI). Other consistently high ranking features were the Red-Green Pigment Index (RGI), Red-Edge Position Linear Interpolation (REP LE). Top unique indices for the 2020-07-02 data included the Structure Insensitive Pigment Index (SIPI3), Browning reflectance index (BRI), whereas the Simple Ratio Greenness Index (GI), Single Band 720 Boochs2 (Boochs2) were unique top predictors in the later 2020-07-09 data. For the full feature ranking results, please refer to Additional file 1: Table S2.

**Table 1:** HTPP features selected through recursive feature elimination to predict YR Features marked in bold are unique to the timepoint

Model trained on data collected on 2020-07-02			Model trained on data collected on 2020-07-09		
Model feature	Average rank across all resamples	Standard deviation	Model feature	Average rank across all resamples	Standard deviation
PSRI	3.10	2.62	RGI	1.40	0.81
PRI2	3.27	2.35	**GI**	3.40	3.17
**SIPI3**	4.63	3.36	PSRI	4.87	2.76
PRI	5.43	4.12	**NRI**	5.87	4.12
REP_LE	5.53	3.34	**Boochs2**	6.87	2.49
RGI	5.77	1.89	DPI	7.00	4.17
**BRI**	10.77	4.44	REP_LE	8.63	4.12
SR9	14.00	6.72	SR10	10.30	4.23
Vogelmann3	17.10	6.82	PRI	12.30	4.69
PRI norm	17.53	9.55	PRI2	13.10	5.36
**LRDSI2**	18.87	10.83	**Datt5**	13.27	10.05
PRI (YR Zheng)	20.97	10.97	DWSI4	14.60	8.32
DD	22.07	16.16	CI	15.03	8.93
DPI	22.27	9.50	Vogelmann3	15.40	5.20
**ARI**	22.57	5.85	**SPVI**	17.07	8.66
**mSR705**	22.93	12.24	ARI2	19.43	6.52
SIPI2	25.63	14.27	PRI (YR Zheng)	20.07	6.76
**mND705**	25.67	14.20	**Boochs**	20.57	8.70
**MTCI**	25.77	15.49	NDVI3	21.43	9.62
**SR7**	26.77	15.03	PRI_norm	22.30	5.07
**D1**	27.23	12.15	**NPQI**	22.77	9.13
mSR	27.77	19.16	**SR5**	25.13	9.02
**NIR Camera BNDVI**	29.20	11.86	**Carter5**	28.33	10.49
**mNDVI**	31.27	15.72	**RGB_Biomass**	29.23	9.48
**MSAVI**	31.63	19.99	DD	30.43	16.17
SR8	32.23	13.74	**EVI**	32.97	10.13
SIPI	32.27	16.97	**MCARI2**	33.43	13.59
**PhRI**	32.30	12.07	NPCI	35.50	12.56
**BGI**	32.73	12.66	**DDn**	35.67	10.10
NDVI3	32.97	17.46	**SRPI**	35.67	11.28
**OSAVI**	33.13	15.26	**ClAInt**	36.07	13.96
ARI2	33.67	11.50	**Sum_Dr2**	36.17	15.92
DWSI4	33.80	17.01	Gitelson	36.63	11.03
mREIP	34.27	10.68	SIPI2	37.17	15.58
CI	36.47	12.66	mREIP	38.23	8.80
**REP_Li**	36.67	11.06	**Datt3**	38.47	13.72
MCARI2_OSAVI2	37.67	19.27	SR8	38.57	8.62
**SAVI**	38.07	15.66	mSR	38.60	16.38
**Carter**	38.17	13.72	SR9	38.87	11.15
SR10	38.47	13.58	MCARI2_OSAVI2	39.07	12.98
Gitelson	38.70	14.70			
NPCI	39.60	14.79			

Variable names highlighted in bold letters denote predictors unqiuely selected through feature elimination at that timepoint

### Accuracy of predicted disease scores

**Fig. 4:**
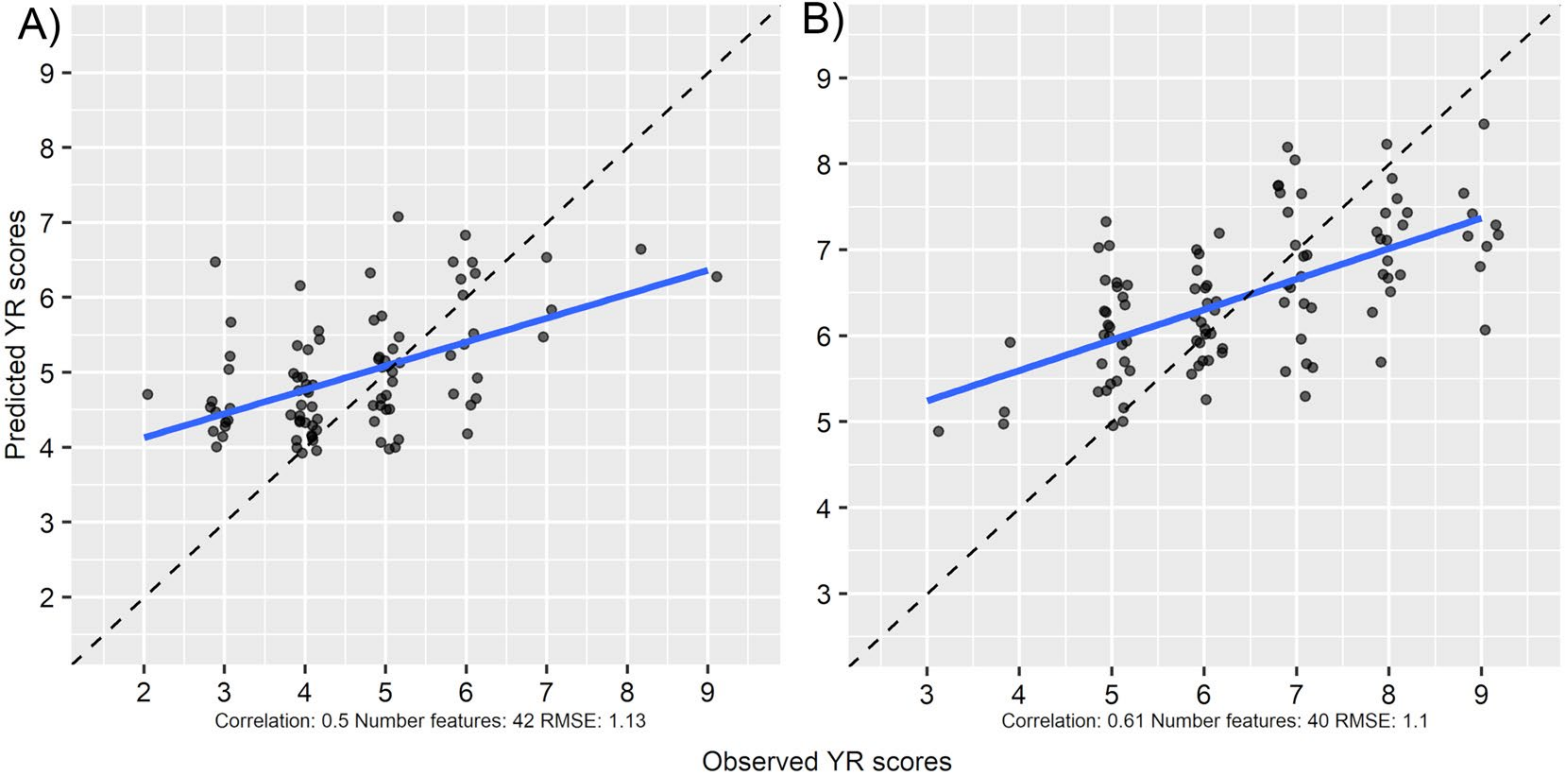
Relation between predicted and observed yellow rust scores based on spectral and image-based predictors. Predicted yellow rust scores from spectral reflectance and image-based predictor data in an independent test set. The dashed line represents the 1:1 line. **a** Disease and HTPP data collected on 2020-07-02 (N = 88), **b** disease and HTPP data collected on 2020-07-09 (N = 78)

**Fig. 5:**
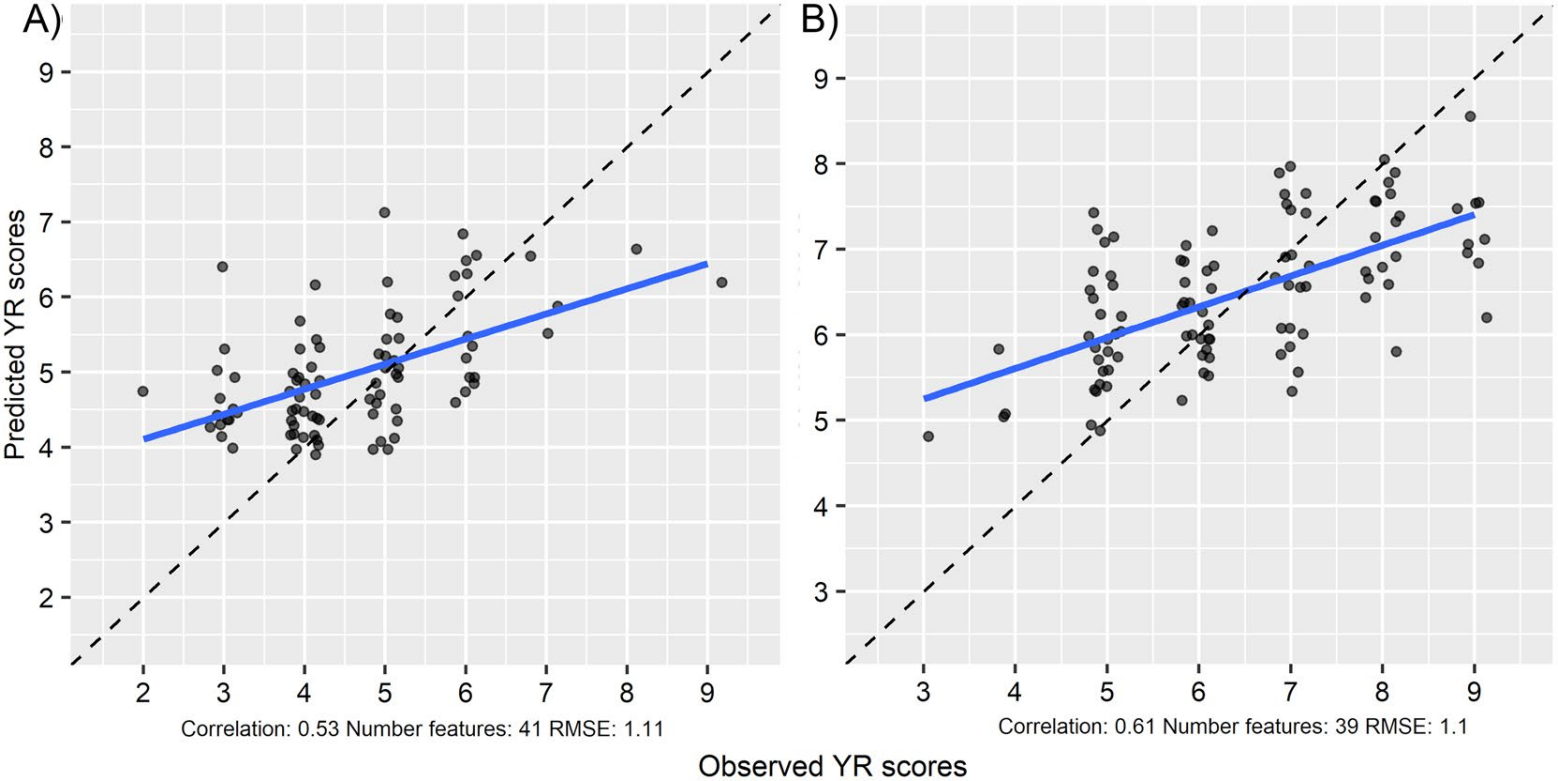
Relation between predicted and observed yellow rust scores using only spectral predictors. Predicted yellow rust scores using only spectral reflectance data in an independent test set. The dashed line represents the 1:1 line. **a** Disease and HTPP data collected on 2020-07-02 (N = 88), **b** disease and HTPP data collected on 2020-07-09 (N = 78)

The top HTPP features identified in the recursive feature elimination and disease observation data were integrated in RF regression models at the two time points (2020-07-02 and 2020-07-09) to predict yellow rust disease scores in the experimental wheat plots. The models were evaluated on a test set of data not used in the feature elimination, where the performance of the models was assessed by computing the RMSE of prediction and correlation between predicted and observed disease scores. This resulted in an estimate of the prediction accuracy of 0.50 and 0.61 for the 2020-07-02 and 2020-07-09 RF models respectively. A linear trend between observed and predicted disease scores was revealed by the plotting the predictions (Fig. 4). The models showed higher separation in their predicted scores when looking at the extreme ends of observed scores. Predictions of intermediate scores showed low separation compared to extreme scores. Removing the more complex image-based predictors from the models resulted in no major change in prediction accuracy when validated on the test set (Fig. 5).

## Discussion

Diseases remain a major yield-limiting factor in wheat cultivation. Thus, breeding for disease resistance remains an important goal in breeding programs, as it remains the most sustainable and cost-effective way of disease control. HTPP methods are then proposed to accelerate the genetic gain achieved in breeding trials. Direct benefits of introducing HTPP methods include increasing the accuracy of selection through less subjective and more repeatable measurements, while indirect benefits include reducing costs associated with phenotyping, which opens the path for allocating more resources to increasing the size of breeding programs [23]. In this vein, we aimed to develop a method to enable rapid, low-cost screening of two diseases in wheat breeding trials, by integrating proximal sensor data with visual disease score data collected under field conditions.

### Low-cost field-based prediction of yellow rust

The correlation analysis (Fig. 2) revealed that the individual predictors derived from the field HTPP data show low to moderate correlation with the observed disease data. This suggests that there are predictor variables in our data which might be used to predict yellow rust scores. This was indeed confirmed when integrating the HTPP and disease data in machine learning models. Using our approach, which combines low-cost sensors with machine learning, we were able to predict yellow rust disease scores at specific time points with an average prediction accuracy of 0.50–0.61 when using a cross-validation approach (Fig. 4). This prediction accuracy presents a positive linear correlation of observed and predicted disease scores, which suggests that our approach shows some success in field-based prediction of yellow rust. However, both models show considerable systematic error, where on average low scores are overestimated and high scores are underestimated. Furthermore, the precision of the predicted values is low. The accuracy and precision of the predictions could perhaps be improved by incorporating spectral data from further into the infrared spectrum (700–1000 nm). For example, a SVI has been previously proposed to measure yellow rust in mid-late stage wheat [10] which relies on spectral data obtained at 860 nm, which is outside of the spectral range at which the spectroradiometer used in this study operates. Further improvement could come in form of replacing or combining the visual scores, used as ground truth data in this study, with more objective digital methods to estimate disease infection, such as destructive sampling and image analysis of diseased leaves, as was done in a similar study of field-based prediction of STB [13]. In that study, the authors reported a prediction accuracy of r = 0.53 when validating their proposed model in an independent test set. The drawback to obtaining disease score data this way, however, is that such a method would be more labor-intensive compared to visual scoring. A similar study aiming to predict yellow rust in field conditions reported prediction accuracies of r = 0.84 using a ground-based hyperspectral-camera based approach [11]. While this is a higher prediction accuracy, hyperspectral imaging sensors are more expensive and more challenging in terms of data analysis, compared to our low-cost approach.

### Evaluation of sensors for prediction

The final step of the analysis was assessing the variable importance in the RF models trained on the complete data sets. The most important predictors were all SVIs sensitive towards changes in leaf pigments (Table 1), such as the plant senescence reflectance index (PSRI) which is designed to track the senescence progress in leaf tissue [24] and photochemical reflectance index (PRI) which is sensitive to changes in xanthophyll pigments in leaf tissue and is used to changes in photosynthetic performance [25]. Other top predictors related to changes in leaf pigments were the Red-Green Pigment Index (RGI) [26], Normalized Photochemical Reflectance Index (PRI_Norm) [27], Normalized-Difference (570/539) Photochemical Reflectance Index (PRI2) [28], Red-edge position linear extrapolation (Rep LE) [29], Greenness Index (GI) [30], Double Peak Index (DPI) [31], Boochs2 [32], and Vogelmann3 [33]. The high importance of indices related to changes in leaf pigments and chlorophyll fluorescence is in line with the effects of yellow rust infection on the leaf. These effects include the degradation of chlorophyll, which leads a reduced photosynthetic efficiency. In addition, the disease symptoms progress into the formation of yellow carotenoid-rich stripes of spores, which give the yellow rust disease its name. Our results mirror an earlier study [9] which found that the PRI, anthocyanin reflectance index (ARI), PSRI and other indices related to changes in leaf pigment concentrations could be used to detect yellow rust-infected leaves in wheat under controlled conditions. Similarly, a different study [10] conducted a field experiment investigating 15 previously published indices and their ability to detect yellow rust, in addition to two modified versions of the PRI and ARI indices. The authors found that the modified three-band PRI and ARI indices were sufficient to detect and measure yellow rust in early-mid growth and mid-late stages respectively. Another study investigated the potential of individual indices to be used for indirect measurements of yellow rust disease severity, and found that PRI, GI were among the better performing indices [34]. PRI also figures in patented methods for predicting yellow rust in wheat crops [35]. The three-band PRI (PRI_YR) index ranked 12th and 17th respectively in the ranking produced by the recursive feature elimination (Table 1), suggesting that it might be used for predicting yellow rust in late-stage winter wheat in combination with other SVIs. The ARI-based index, developed in the same study as above, for detection and measuring of yellow rust in late-stage winter wheat was not used in this study, as our spectroradiometer did not make measurements in the spectral bands required to estimate the index. In contrast, the predictor ranking revealed that the previously developed Yellow Rust Index [20] did not rank among the selected predictors, suggesting it has limited use in predicting yellow rust scores in late/stage winter wheat. The predictors selected in the final models based on the RFE included the digital green biomass estimates (RGB Biomass) and NDVI estimates (NIR BNDVI) from the imaging sensors, although not among the top predictors. The relatively low importance of NDVI could be explained by its lack of specificity when detecting stress. Furthermore, it should be added that the data processing of the imaging data is more time-intensive and laborious compared to working with the spectral data. To further investigate whether these image-based predictors were worth the effort, the final prediction models were retrained excluding them. The resulting models were evaluated on the independent test set where no major decrease in prediction accuracy was observed (Fig. 5). While this does not disqualify the imaging sensors per se, it does suggest their use needs rethinking for them to be a worthwhile addition in proximal phenotyping of yellow rust. For example, a future study could investigate replacing the green biomass estimates from the RGB camera with detecting and quantifying rust lesions on diseased leaves in the top-down images. One approach to accomplish this could be to use deep learning-based models, such as convolutional neural networks, to extract disease severity metrics from the image data. However, this would require a much larger image dataset in the order of 10,000 images compared to the datasets utilized in this study, which count around 400–500 images per timepoint [36].

## Conclusion

The results published in this study suggest that spectral sensors can be used in combination with machine learning to rapidly predict yellow rust scores late in the wheat growing season with a moderate prediction accuracy. Improvements to the collection of disease data can help raise the accuracy and precision of the predictions. A suggestion for future work is to investigate using more objective analysis methods for collecting the disease data, such as sampling and image-analysis of diseased leaves, with the aim of improving the quality of the disease data over subjective visual assessments. Furthermore, a follow-up study could be conducted with an appropriate experimental field design, with the aim to investigate the performance of our method in its intended application of gathering data for selections in un-phenotyped wheat populations, and whether the predicted scores can be used in a genetic analysis of disease resistance.

## Supplementary Information

**Additional file 1: Table S1.** Spearman’s correlation analysis between yellow rust disease scores and model predictor variables derived from high-throughput phenotyping (HTPP) data collected at two dates under field conditions.

**Additional file 2: Table S2.** Full ranking of high-throughput phenotyping features of based on results of recursive feature elimination (RFE).

## Data Availability

The datasets used and/or analysed during the current study are available from the corresponding author on reasonable request.
